# Why genes extending lifespan in model organisms have not been consistently associated with human longevity and what it means to translation research

**DOI:** 10.4161/15384101.2014.950151

**Published:** 2014-10-30

**Authors:** João Pedro de Magalhães

**Affiliations:** Integrative Genomics of Ageing Group; Institute of Integrative Biology; University of Liverpool; Liverpool, UK

**Keywords:** aging, genetics, GWAS, humans, longevity, model organisms

## Abstract

A recent paper by Deelen et al. (2014) in *Human Molecular Genetics* reports the largest genome-wide association study of human longevity to date. While impressive, there is a remarkable lack of association of genes known to considerably extend lifespan in rodents with human longevity, both in this latest study and in genetic association studies in general. Here, I discuss several possible explanations, such as intrinsic limitations in longevity association studies and the complex genetic architecture of longevity. Yet one hypothesis is that the lack of correlation between longevity-associated genes in model organisms and genes associated with human longevity is, at least partly, due to intrinsic limitations and biases in animal studies. In particular, most studies in model organisms are conducted in strains of limited genetic diversity which are then not applicable to human populations. This has important implications and, together with other recent results demonstrating strain-specific longevity effects in rodents due to caloric restriction, it questions our capacity to translate the exciting findings from the genetics of aging to human therapies.

The recent work by Deelen et al.^1^ is the largest genome-wide association study (GWAS) for human longevity to date with over 20000 long-lived individuals between meta-analysis and validation. Apart from the well-known association on the *TOMM40*/*APOE*/*APOC1* locus, Deelen et al. also found a new association on chromosome 5q33.3 which might be due to a long noncoding RNA (lincRNA). This is an exciting finding for many reasons: The 5q33.3 locus appears to be associated with survival beyond 90 y and, while associated with blood pressure in middle age, at older ages additional processes appear to influence its relation to longevity. The fact that this is a lincRNA is also exciting given the recent interest and the regulatory functions of these non-coding genes of which we know so little about. Changes in expression with age in lincRNAs of unknown function have also been observed in the rat brain using RNA-seq.^2^ Therefore, functional studies are warranted of this relatively new layer of genomic regulation.

A new locus associated with longevity is an important breakthrough, but equally striking are the loci not associated with longevity. According to the GenAge database of aging-related genes,^3^ >1,000 genes have been associated with longevity and/or aging in model organisms, including >100 in mice of which 51 have life-extending effects. None of these was associated with longevity in this latest, large study. This does not come as a surprise since recent large-scale studies of human longevity have invariably been restricted to finding statistically significant associations in the *TOMM40*/*APOE*/*APOC1* locus.^4,5^ Even when using more relaxed significance criteria, results are disappointing: For example, in the 281 genetic variants Sebastiani et al.^4^ found to discriminate between centenarians and controls, 4 genes associated with a shorter lifespan in mice are present (*APOE*, *LMNA*, *SOCS2* and *SOD2*), yet not a single gene was found by Sebastiani et al. with known life-extending effects in mice. Although candidate gene studies have found a few associations related to specific longevity-associated genes such as *IGF1R*^6^ and *FOXO3A*,^7^ these have not been validated in the larger GWAS^1,4^ or by and large in other populations.^5^ So of the 51 genes associated with life-extension in mice, why have these not been convincingly associated with human longevity?

There are several possible explanations. Missing heritability has been observed in other complex traits and diseases.^8^ Even for highly heritable traits like height, many genes with small effects are usually found.^8^ As such, many genes with small effect sizes might contribute to human longevity and these are difficult to detect even in large studies. Nonetheless, because, for instance, in the case of height over 50% of the phenotype can be explained by common variants,^8^ our progress in understanding the genetic determinants of human longevity has arguably been disappointing even compared to other complex traits. As Deelen et al. point out, human longevity association studies have intrinsic limitations, such as the lack of appropriate controls in that among the younger individuals used as controls many will turn out to also be long-lived and the relatively modest heritability of longevity (∼25% whereas the heritability of, say, height is ∼80%). Another hypothesis is that common genetic variants in human populations in genes associated with aging in model organisms are not functionally relevant in context of longevity. Most aging-related genes in mice were derived from knockouts or overexpression manipulations that have strong molecular effects on the gene in question that may not have common equivalents in the general human population. That said, and while it is plausible that human genome resequencing studies will reveal new loci with strong longevity effects, the few studies conducted thus far in human cohorts with mutations in genes associated with longevity in model organisms do not suggest strong effects on longevity. For example, disruption of the growth hormone receptor (GHR) in mice extends lifespan >40 %,^9^ yet GHR deficiency in humans does not result in reduced mortality, even if it appears to protect from cancer.^10^

A more disconcerting interpretation is that the lack of correlation between longevity-associated genes from model organisms and genes associated with human longevity is, at least partly, due to intrinsic limitations and biases in animal studies. Model organisms have contributed tremendously to research on aging and most translational research on aging is based on genetic discoveries in these,^11^ but they also have major drawbacks.^12,13^ One key weakness is that, by and large, longevity studies in model organisms are performed on strains of limited genetic diversity. The roundworm *C. elegans*, for example, has proven to be the most popular model system in the genetics of aging with >700 genes in GenAge, yet it is remarkable that nearly all of these studies have been conducted in the N2 strain. Mouse studies usually employ specific strains with C57BL/6 as a popular choice in longevity studies, yet all strains have their own particular phenotypes and diseases. Indeed, strain-specific effects of longevity-associated genes are known. For example, Ames dwarf mice due to a mutation in *Prop1* are considerably long-lived,^14^ yet the effects of deletion of *Prop1* are strongly influenced by genetic background and in some cases can result in respiratory distress symptoms and even neonatal death.^15^

The reduced genetic and environmental diversity of model systems also fails to capture pleiotropic effects of aging-related pathways. For example, low IGF1 signaling has been consistently associated with life-extension in rodents and with cancer protection,^16^ and indeed Ames dwarf mice have low circulating IGF1 levels, yet low IGF1 can also be detrimental having been associated with, for instance, sarcopenia and cognitive dysfunction.^17,18^ In humans, findings are contradictory concerning the association between IGF1 levels and survival.^19,20^ Beneficial effects of low IGF1 on human survival seem to be mostly observed in individuals susceptible to malignancy,^20^ and it is noteworthy that traditional mouse strains like C57BL/6 die primarily of cancer.

The fact that the life-extending effects of caloric restriction (CR) in mice are strain-specific^21^ adds weight to the idea that having a whole field based on findings that come primarily from clones is problematic. Not surprisingly, when wild-derived, genetically heterogeneous mice are put on CR very modest effects are observed, appearing to be beneficial to some animals but not to others.^22^ Recent results from rhesus monkeys^23,24^ showing much more modest effects of CR than observed in mice or rats further emphasize concerns regarding studies in short-lived models of limited or no genetic diversity. Of course this touches on another potential problem which is that pathways that extend lifespan in short-lived organisms may not work the same way in long-lived ones, a problem pointed out long ago by researchers in the field.^25^

This discussion has important practical implications because if the gene manipulations identified in model organisms to modulate aging and extend longevity are only beneficial to a small percentage of individuals, then this questions our capacity to translate findings from the biology and genetics of aging. I do not think that this excludes potential applications concerning age-related diseases, particularly cancer,^16^ but it suggests that systematic biases in animal longevity studies could considerably decrease our ability to translate the extraordinary findings in model organisms to extend human life and preserve health, arguably the goal of biogerontology. The NIH interventions testing program employs genetically heterogeneous mice to minimize such effects,^26^ but given costs and funding climate validation of the impressive results on the genetics of aging in additional mouse strains is unlikely to be pursued in the foreseeable future. Therefore, of the 51 gene manipulations extending lifespan in mice, how many would still extend lifespan in genetically heterogeneous mice and by how much? How many would be detrimental? When considering potential applications of the genetics of aging one should keep in mind that these have not been replicated in humans and that even in model organisms these are derived from a very small selection of clones that do not represent the whole species.
